# Single-cell transcriptomics reveals neural stem cell trans-differentiation and cell subpopulations in whole heart decellularized extracellular matrix

**DOI:** 10.52601/bpr.2024.240011

**Published:** 2024-08-31

**Authors:** Xiaoning Yang, Yuwei Zhao, Wei Liu, Zhongbao Gao, Chunlan Wang, Changyong Wang, Siwei Li, Xiao Zhang

**Affiliations:** 1 Beijing Institute of Basic Medical Sciences, Beijing 100850, China

**Keywords:** Single-cell transcriptomics, Neural stem cell, Trans-differentiation, Decellularized extracellular matrix, Cardiac lineage cells

## Abstract

The whole heart decellularized extracellular matrix (ECM) has become a promising scaffold material for cardiac tissue engineering. Our previous research has shown that the whole heart acellular matrix possesses the memory function regulating neural stem cells (NSCs) trans-differentiating to cardiac lineage cells. However, the cell subpopulations and phenotypes in the trans-differentiation of NSCs have not been clearly identified. Here, we performed single-cell RNA sequencing and identified 2,765 cells in the recellularized heart with NSCs revealing the cellular diversity of cardiac and neural lineage, confirming NSCs were capable of trans-differentiating into the cardiac lineage while maintaining the original ability to differentiate into the neural lineage. Notably, the trans-differentiated heart-like cells have dual signatures of neuroectoderm and cardiac mesoderm. This study unveils an in-depth mechanism underlying the trans-differentiation of NSCs and provides a new opportunity and theoretical basis for cardiac regeneration.

## INTRODUCTION

Among the natural materials, the decellularized matrix has been widely used for tissue reconstruction with the closest components and ultrastructural properties to the extracellular matrix (Bonnans *et al.* 2014; Kim *et al.* 2017). A whole organ or tissue decellularized matrix could provide a unique microenvironment for stem cells and direct cell behaviors (Agmon and Christman 2016; Han *et al.* 2019). Previous studies have confirmed whole organ or tissue decellularized matrix, derived from the lung (Shojaie *et al.* 2015), liver (Lang *et al.* 2011), kidney (Zhou *et al.* 2020), heart (Ng *et al.* 2011), and so on, could dictate tissue-specific cell differentiation and maturation of stem and progenitor cells (Wang *et al.* 2021b).

Myocardial infarction is a major cause of death and disability worldwide (Gaudron *et al.* 1993). Cardiac engineering provides a meaningful strategy to regenerate myocardium and whole heart decellularized matrix has received much attention (Bejleri and Davis 2019; De Santis *et al.* 2021; Ng *et al.* 2011; Pagliarosi *et al.* 2020; Pati *et al.* 2014). Abundant evidence has shown that the microenvironment of the whole heart acellular matrix could affect cell behavior and fates of stem cells (Jang *et al.* 2017; Ronaldson-Bouchard *et al.* 2018; Sullivan *et al.* 2014; Zhang *et al.* 2012). Human embryonic stem cells (hESCs), induced pluripotent stem cells (iPSC), and mesendodermal cells derived from hESCs in the heart decellularized matrix showed significant upregulated expression of cardiac markers upon differentiation (Goldfracht *et al.* 2019; Hochman-Mendez *et al.* 2020; Li *et al.* 2017; Ng *et al.* 2011). We wondered how the whole heart acellular matrix has an effect on ectodermal stem cells NSCs, in contrast with those stem cells possessing the innate capacity to differentiate into cardiomyocytes. Our earlier findings have shown that NSCs exhibited a tendency of trans-differentiating into cardiac-related lineages, illustrating whole heart acellular matrix possessed the memory function and provided an ideal niche for the cardiac-specific trans-differentiation of NSCs (Wang *et al.* 2021a). but little is known about the molecular mechanism underlying trans-differentiation of NSCs.

**Figure 1:**
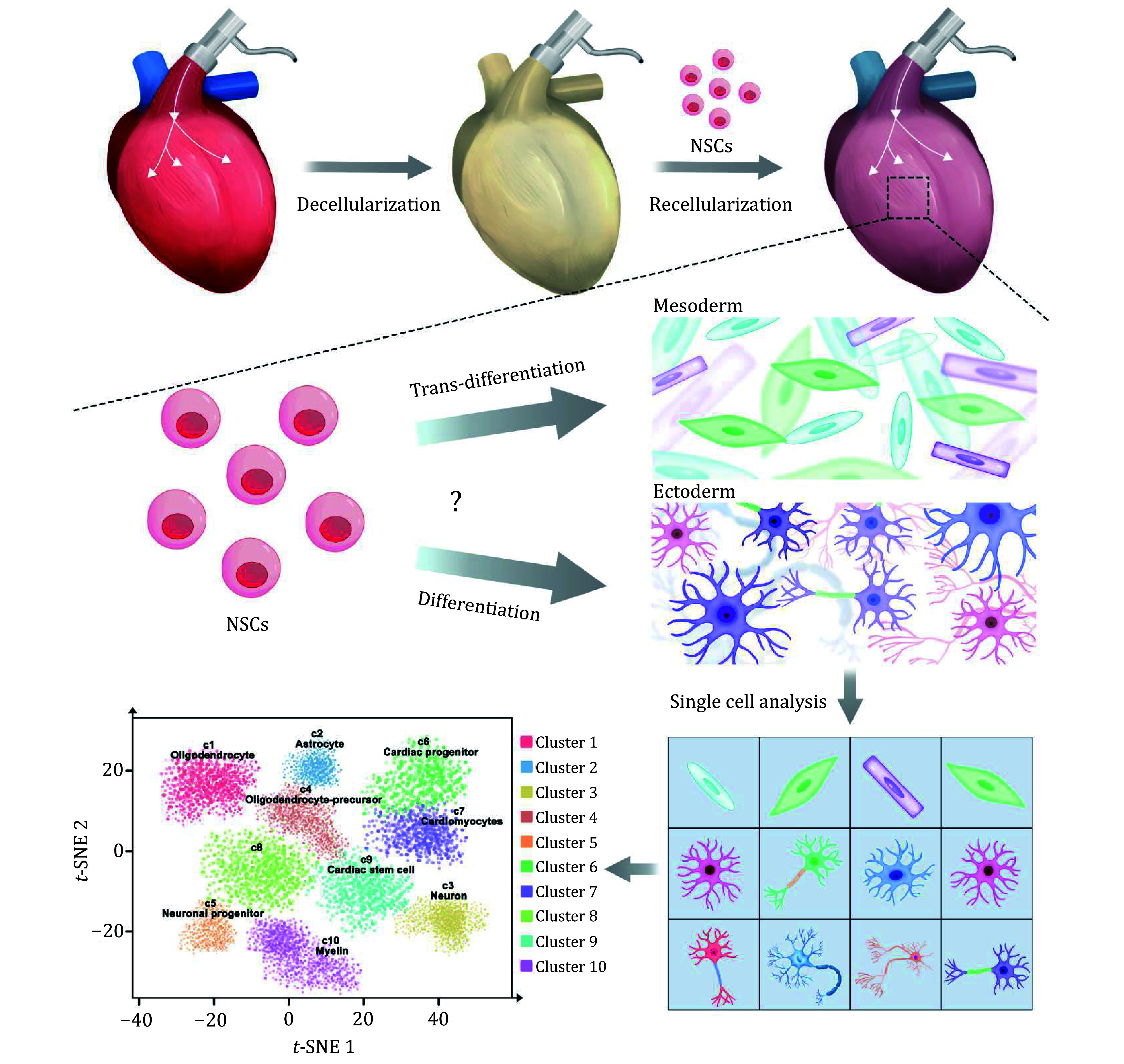
Schematic representation of single-cell transcriptomic profiling in recellularized heart construct with NSCs

To deeply understand how the whole heart acellular matrix directs trans-differentiation of NSCs, in this study, we used single-cell RNA-seq technology to systematically analyze the cell types and molecular characteristics in recellularized heart construct with NSCs (Fig. 1). We found that the recellularized construct had obvious characteristics of myocardial lineage differentiation while maintaining neural lineage differentiation. Our findings show that tissue-specific microenvironment can provide the potential to determine the fate of stem cells originating from different germ layers, which provides novel insights into the trans-differentiation of NSCs and lays the foundation for the application of whole heart decellularized matrix in regenerative medicine.

## EXPERIMENTAL SECTION

### Preparation of whole heart decellularized matrix

Animal organs were procured in compliance with approved animal protocols and institutional guidelines. The Sprague-Dawley rat donors (260–280 g) were pre-treatment with barbital sodium for anesthesia and then administered a dose of heparin (2.0 U/g of body weight) into the post cava. The heart was isolated and stored at −80 °C. Prior to decellularization, the rat hearts were thawed at room temperature and connected to a perfusion pump (Leifu) at a rate of 3 mL/min. The hearts were sequentially perfused with distilled water for 15 min, 1% sodium dodecyl sulfate (SDS, Invitrogen) for 12 h, 1% Triton-X100 (Sigma) for 30 min, phosphate-buffered saline (PBS) for at least 72 h to eliminate residual Triton-X100 and SDS, and high-glucose Dulbecco’s Modified Eagle Medium (H-DMEM, Gibco) overnight before recellularization.

### Histology and immunofluorescence

The native hearts, decellularized heart scaffolds and recellularized constructs were embedded in paraffin and sectioned. The paraffin-embedded sections were stained by hematoxylin and eosin (H&E) following the manufacturer’s protocols to evaluate effects of decellularization and recellularization. Immunostaining of recellularized constructs was conducted with antibodies of neural lineage marker MAP2 and GFAP (Invitrogen) and cardiac lineage marker α-actinin (Invitrogen) following the manufacturer’s protocols. All secondary antibodies were purchased from Invitrogen. Images were taken with a Leica DMRA microscope.

### Scanning electron microscopy

The native hearts and decellularized extracellular matrix were cut into small blocks (8 mm^3^), fixed with 2.5% glutaraldehyde for 2 h at room temperature, and then washed three times with PBS (5 min each). Subsequently, they underwent sequential dehydration using 30%, 50%, 70%, 90%, and 100% ethanol, followed by freeze-drying using a lyophilizer. The samples were then coated with 2 µm AuPd using a sputter coater system (sputter module 108auto, Cressington Scientific, Watford, UK). Finally, images were taken using a Jeol6335F field emission SEM with a backscatter detector (Nova200 NanoLab).

### Cell culture

NSCs were obtained from the rat cortex on embryonic day 14 (E14) and maintained in proliferation medium, consisting of DMEM/F12 (1:1; Invitrogen, Carlsbad, CA) supplemented with 20 ng/mL basic fibroblast growth factor (Invitrogen), 20 ng/mL epidermal growth factor (Invitrogen), and 2% B27 supplements (Invitrogen). Passage of the cells was performed every three days.

### Recellularization in decellularized heart matrix

Approximately 3 × 10^7^ NSCs were seeded into the decellularized heart matrix by injecting them five times using 300 µL of basic differentiation media (H-DMEM + 20% FBS). To ensure a stable interaction of the cells with the matrix, we stopped perfusing the recellularized heart after seeding to prevent cell washout from the reseeded decellularized matrix. During the initial two days, the recellularized construct was maintained in a two-dimensional non-perfused tissue-culture dish. Following this static culture period, the recellularized constructs were integrated into a perfusion system and submerged in the medium. After an additional 24 hours, the recellularized construct was raised and continuously perfused with a medium. The medium was refreshed every 48 hours.

### Dissociating the recellularized constructs for single-cell suspension

The recellularized constructs which had been perfusion cultured for 14 days were collected and washed three times in 1× PBS, and then dissociated in 5 mL of 0.25% trypsin for approximately 45 min to detach the cells from the scaffold. The cell suspension was filtered through the strainer with the diameter of 40 μm to create a single-cell suspension later. Cells were collected by centrifugation at 300 relative centrifugal force for 5 min and resuspended in 500 μL of PBS (0.04% BSA). The single-cell suspensions were resuspended at a concentration of 800–1000 cells/μL. Next, the cell viability was assessed by Trypan blue staining (typically 85%–95% viable) and counted using a cell counter.

### Capturing of single cells and preparation of complementary DNA

Single-cell suspensions were loaded onto 10X Genomics Single Cell 3' Chips along with the reverse transcription (RT) master mix, following the manufacturer's protocol for the Chromium Single Cell 3’ Library (10XGenomics; PN-120233) to generate single cell gel beads in emulsion (GEMs). Each sample was sequenced in a single lane using the Illumina HiSeq2500 in Rapid Run Mode using a paired-end flow cell as previously described.

### Single-cell RNA seq data

Sequenced data was analyzed using the Seurat package in R for single-cell analysis. Cells were processed via the Seurat workflow to remove doublets and unwanted sources of variation (Satija *et al.* 2015). We filtered the data to exclude low-quality cells with <200 expressed genes and low-expressed genes that are expressed in less than three cells from all downstream analyses. Cells were clustered using *t*-SNE and *k*-means. Cells were projected onto a 2D embedding using *t*-Distributed Stochastic Neighbor Embedding (*t*-SNE) with cell loadings associated with principal components utilizing all expressed genes as input. *K*-means clustering was run to group cells for the clustering analysis (McDavid *et al.* 2013). *K* = 10 was selected based on the sum of the squared error scree plot. After clustering cells, the differential gene expression between different cell clusters was analyzed using the Seurat software package. We used the FindAllMarkers function to compare the expression levels of a cell cluster to all other cell clusters in that sample, and obtained a list of differentially expressed genes for that cell cluster.

### Identification of cluster-specific genes and marker-based classification

To identify the cell type of each cluster, the specific markers of the potential cell types were initially collected, which were then compared with the differently expressed genes of each cluster. For the genes that are enriched in a specific cluster, the mean expression of each gene was calculated across all cells in the cluster. Next, each gene from the cluster was compared to the median expression of the same gene from cells in all other clusters. For hierarchical clustering, the pair-wise correlation between each cluster was calculated, and the centered expression of each gene was used for visualization by heat map. Genes satisfying *p* < 0.01 were considered statistically significant. Gene Ontology enrichments among cluster enriched, differential genes were computed using Metascape.

### GO and KEGG pathway enrichment

GO and KEGG enrichment analysis of differentially expressed gene sets were implemented in the GOseq R and KOBAS 3.0 package, respectively. GO terms and KEGG pathways with adjusted P-value below 0.05 were considered as significantly enriched by differentially expressed genes.

## RESULTS

### Preparation and characterization of whole heart decellularized extracellular matrix

**Figure 2:**
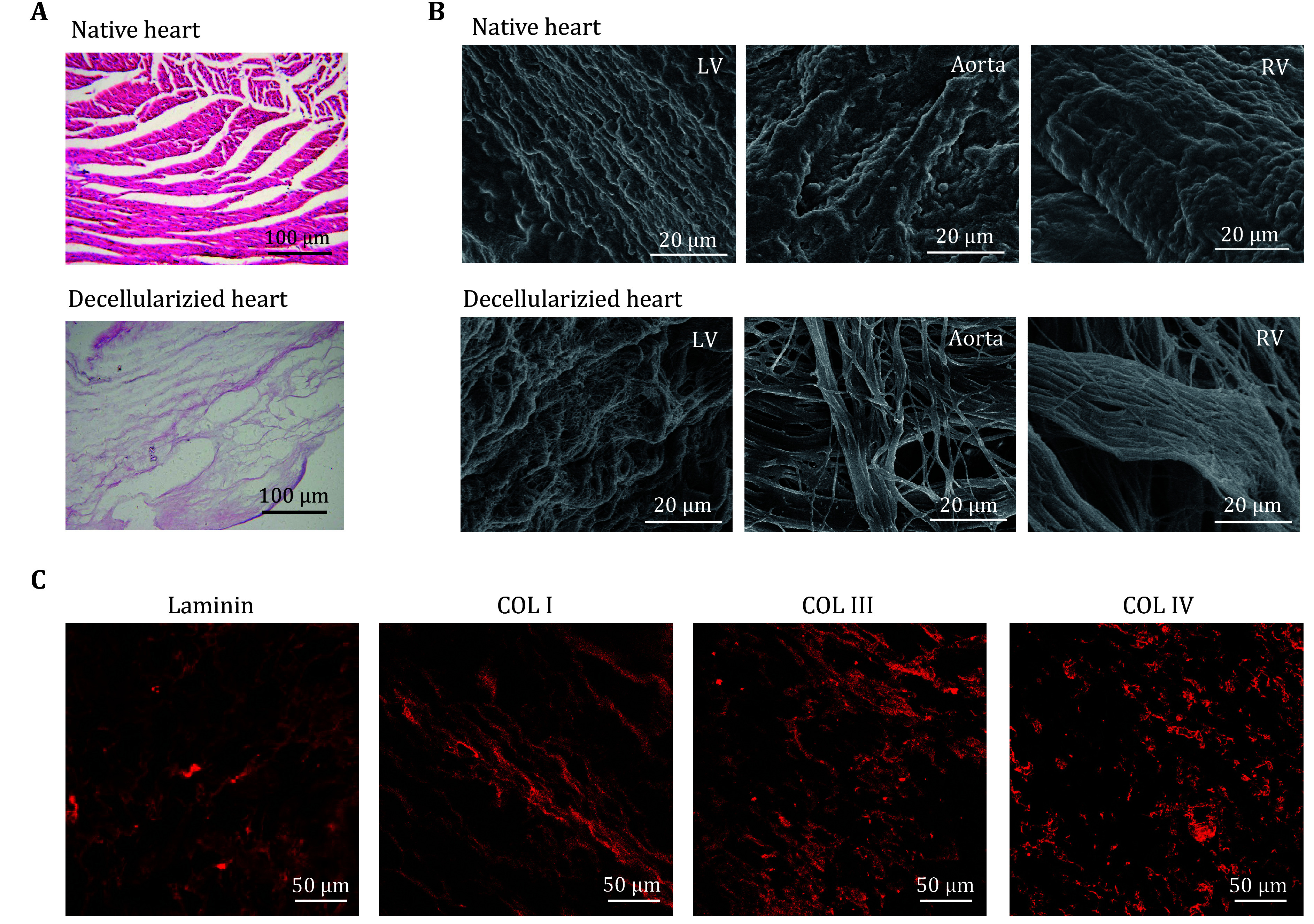
Characterization of whole heart decellularized extracellular matrix. **A** H&E staining of the native heart and decellularized heart matrix. **B** SEM images of the native heart and decellularized heart matrix. **C** Immunostaining of decellularized heart with anti-laminin, anti-Collagen I, anti-Collagen III, anti-Collagen IV antibodies

The whole heart decellularized extracellular matrix was prepared by coronary perfusion of rat hearts. As shown in Fig. 2A, H&E staining images demonstrated cellular content and nuclei were removed. As shown in Fig. 2B, a scanning electron microscope (SEM) showed the decellularized matrix maintained its ultrastructure and remained intact. The decellularized matrix possessed a fibrous network and the ribbon-shaped fibres with random arrangement were identified as attached glycosaminoglycans, forming a porous 3D microenvironment. Furthermore, as shown in Fig. 2C, immunofluorescent staining revealed the presence of ECM components such as collagen I, collagen III, collagen IV, and laminin in the decellularized matrix, maintaining a porous structure and oriented distribution. These results indicated that the whole heart decellularized matrix preserved the structure and membrane components of the ECM of the native heart.

### NSCs Recellularization and differentiation in whole heart decellularized matrix

**Figure 3:**
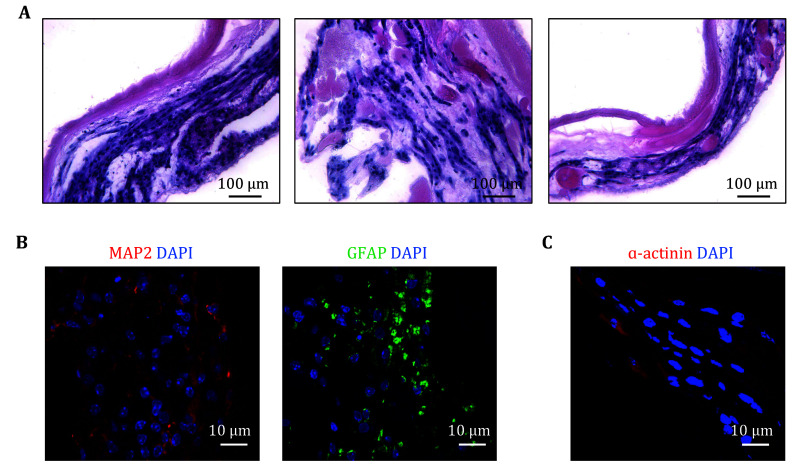
Recellularization and characterization of NSCs in whole heart decellularized matrix. **A** H&E staining of recellularized constructs under the perfusion culture on day 14 post recellularization. **B**, **C** Immunofluorescent staining of recellularized constructs with DAPI, neural lineage specific markers MAP2 and GFAP (**B**), and cardiac lineage specific markers ɑ-actinin (**C**) on day 14 post recellularization

To investigate whether NSCs could be reprogrammed into non-neural lineages through stimulation from tissue-specific microenvironments, approximately 3 × 10^7^ NSCs were injected into the decellularized heart matrix. As shown in Fig. 3A, H&E staining images showed that NSCs spread along the matrix on day 14 of perfusion culturing and some cells exhibited cardiac-like spindle appearance. In addition, the differentiation of NSCs in the decellularized heart matrix was analysed. Immunofluorescent images of MAP2 and GFAP showed the presence of oligodendrocytes and neurons, suggesting that NSCs differentiated into the neural lineage (Fig. 3B). Previous reports have suggested that NSCs could be directly reprogrammed to non-neural cell types under specific conditions (Galli *et al.* 2000; Rietze *et al.* 2001). So, we studied whether heart decellularized heart matrix could direct the lineage commitment of NSCs to resident cell types. The immunofluorescent images of α-actinin showed that some cardiac lineage cells existed (Fig. 3C). These results indicated that the majority of NSCs in whole heart decellularized matrix differentiated into neural-like cells. Besides, NSCs exhibited a tendency to trans-differentiating into cardiac-like cells.

### Single-cell RNA-seq map cell diversity of recellularized constructs

**Figure 4:**
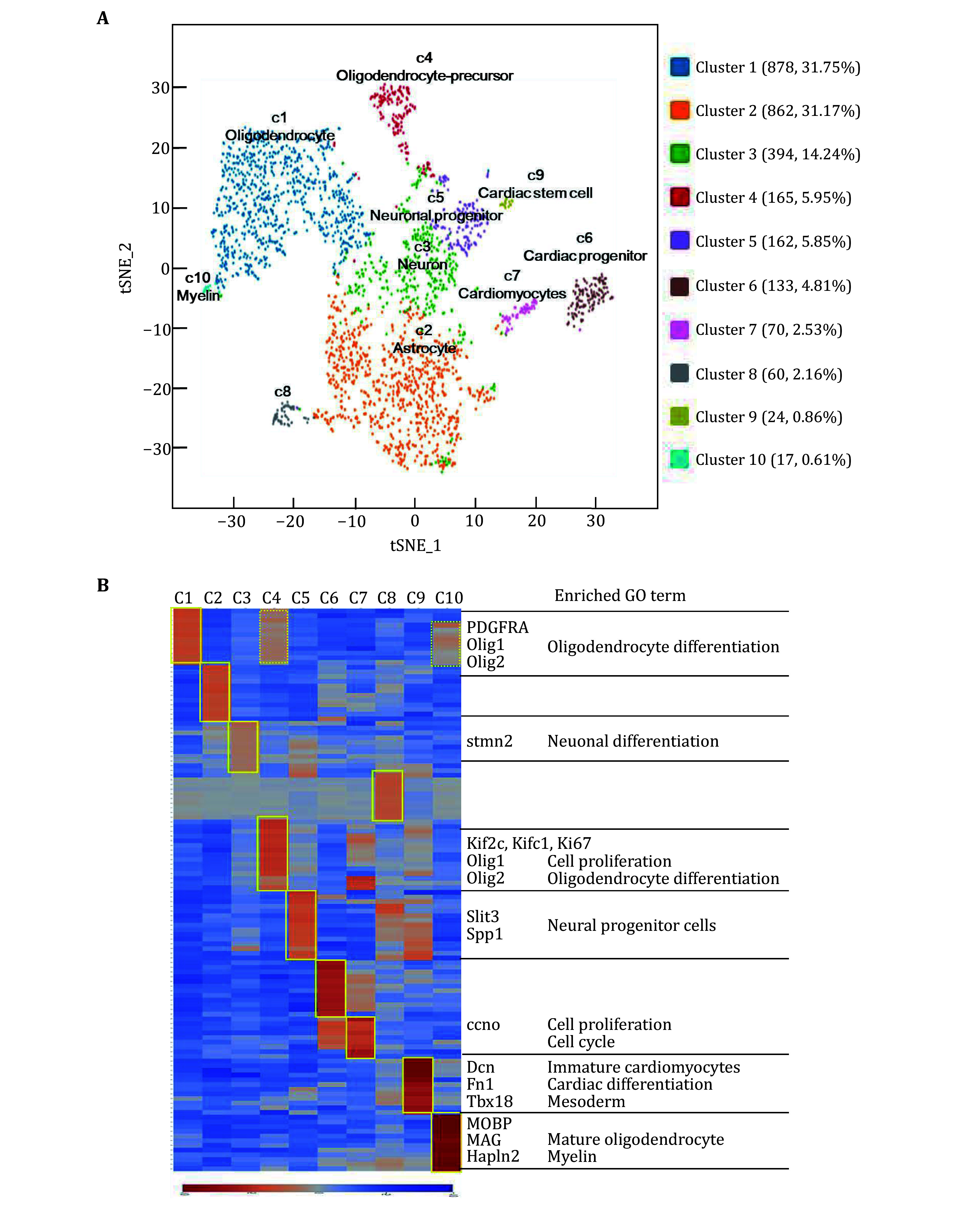
Single-cell sequencing analysis demonstrated the development of cell types in recellularized construct on day 14. **A** tSNE plot of single-cell mRNA sequencing data from the recellularized construct. **B** Heat map shows blocks of genes enriched in each cell type. Specific genes related to each type are highlighted with enriched gene ontology terms (right)

To analyse the molecular features of the cells in the recellularized construct on day 14 post recellularization, we digested the recellularized construct to obtain single-cell suspension and performed single-cell sequencing. 2765 single cells were obtained and principal component analysis (PCA) of read counts was conducted with dimensionality reduction using *t*SNE (Fig. 4A, supplementary Fig. S1). Clustering all cells from the recellularized construct produced ten main transcriptionally distinctive populations. We systematically compared the differentially expressed gene signatures for each cluster and identified the cell-type specific markers (Fig. 4B). We were able to define the identities of 9 clusters, c1, c2, c3, c4, c5, c6, c7, c9 and c10. Specifically, c1 (878 cells, 31.75%) was primarily composed of cells expressing genes of oligodendrocytes, including canonical markers Olig1 and Olig2. C4 (165 cells, 5.95%) was enriched with cells expressing oligodendrocyte-precursor marker (PDGFRA) and proliferative markers, including MKi67, Kif2c and Kifc1, thus c4 was classified as an oligodendrocyte-precursor-like identity. C10 (17 cells, 0.61%) had a clear mature oligodendroglia signature and was enriched with markers of myelin, including Mobp, Mag and Mog. C2 (862 cells, 31.17%) was mainly composed of cells expressing genes of astrocytes, including canonical markers GFAP and Gja1. C3 (394 cells, 14.24%) had a clear neuronal signature and was enriched with markers of neurons (Stmn2, Gad1). C5 (162 cells, 5.85%) contained progenitors of NSCs origin, showing a high expression of neural progenitor specific genes (Runx2, slit3). C6 (133 cells, 4.81%) was composed of cells expressing mature cardiomyocytes specific genes, including TNNi3 and Myh7. C7 (70 cells, 2.53%) was mainly composed of cells expressing cardiomyocyte markers (TNNi3, Myh7) and proliferative markers (Ccno). C9 (24 cells, 0.86%) had a clear signature of cardiac stem cells expressing Tbx18, GATA4, GATA6 and Nkx2-5.

### Single-cell transcriptome analysis dissects multi-lineage communication and cellular heterogeneity in recellularized constructs

**Figure 5:**
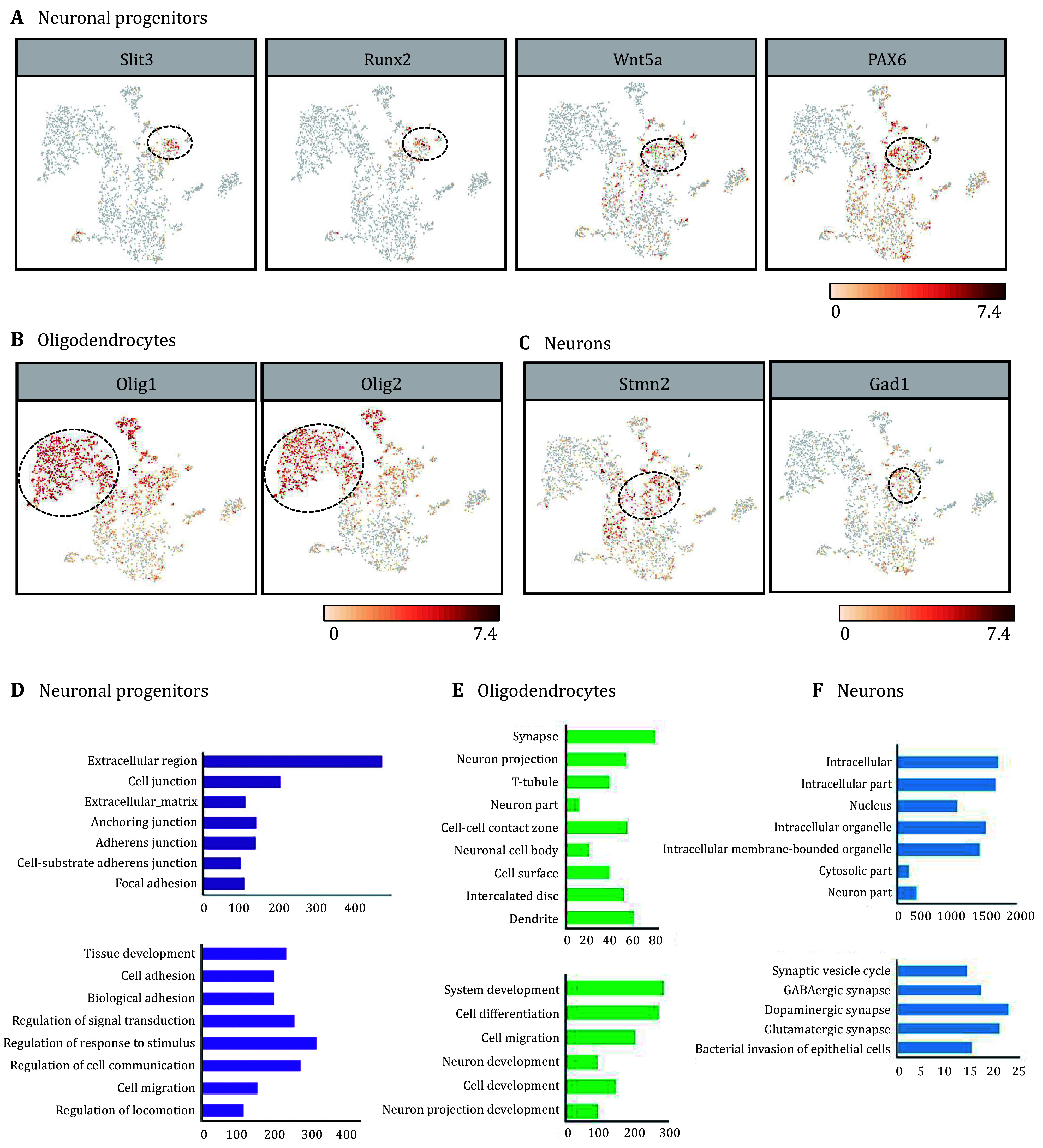
Recellularized construct contained neural cells. **A**-**C** The specific markers were specifically expressed in neural progenitors (c5) (**A**), Oligodendrocytes (c1) (**B**) Neurons (c3) (**C**). **D**-**F** GO analysis of the differently expressed genes in c5, c1 and c3. The section of cellular component and biological process (**D**) in c5; The section of cellular component and biological process (**E**) in c1; The section of cellular component and biological process (**F**) in c3

C1, c3 and c5 were identified as various types of neural lineage cells. C5 was identified as neural progenitors with molecular signatures of slit3, Runx2, and Wnt5 and Pax6 (Fig. 5A). As shown in Fig. 5D, Gene ontology (GO) analysis showed the differently expressed genes in c5 showed significant enrichment in cellular component, including extracellular region and cell junction, and biological process including regulation of cell communication and response to stimulus. These results demonstrated that highly biocompatible extracellular matrix maintained NSCs survival and stemness through interactions of cells-matrix and cell-cell, which is consistent with previous reports that extracellular matrices contain various cell growth factors and support the proliferation, differentiation and migration of NSCs (Prest *et al.* 2018; Wang *et al.* 2013). C1 was identified as oligodendrocytes with molecular signatures of Olig1 and Olig2 (Fig. 5B). As shown in Fig. 5E, the differently expressed genes showed significant enrichment in cell−cell contact zone, neuron projection and system development, demonstrating the function of oligodendrocytes in protecting neural system and aiding signal transduction (Kuhn *et al.* 2019). C3 was identified as neurons with molecular signatures of Stmn2 and Gad1 (Fig. 5C). As shown in Fig. 5F, the differently expressed genes showed significant enrichment in the intracellular part, indicating enhanced intracellular communication, which plays a crucial role in neural development (Sheikh *et al.* 2019). Besides, various types of synapses were enriched, such as GABAergic synapse, Dopaminergic synapse and Glutamatergic synapse, which showed NSCs differentiated into functional neurons. These results suggested that heart decellularized extracellular matrix can be used as bioactive scaffolds to support the differentiation of NSCs.

**Figure 6:**
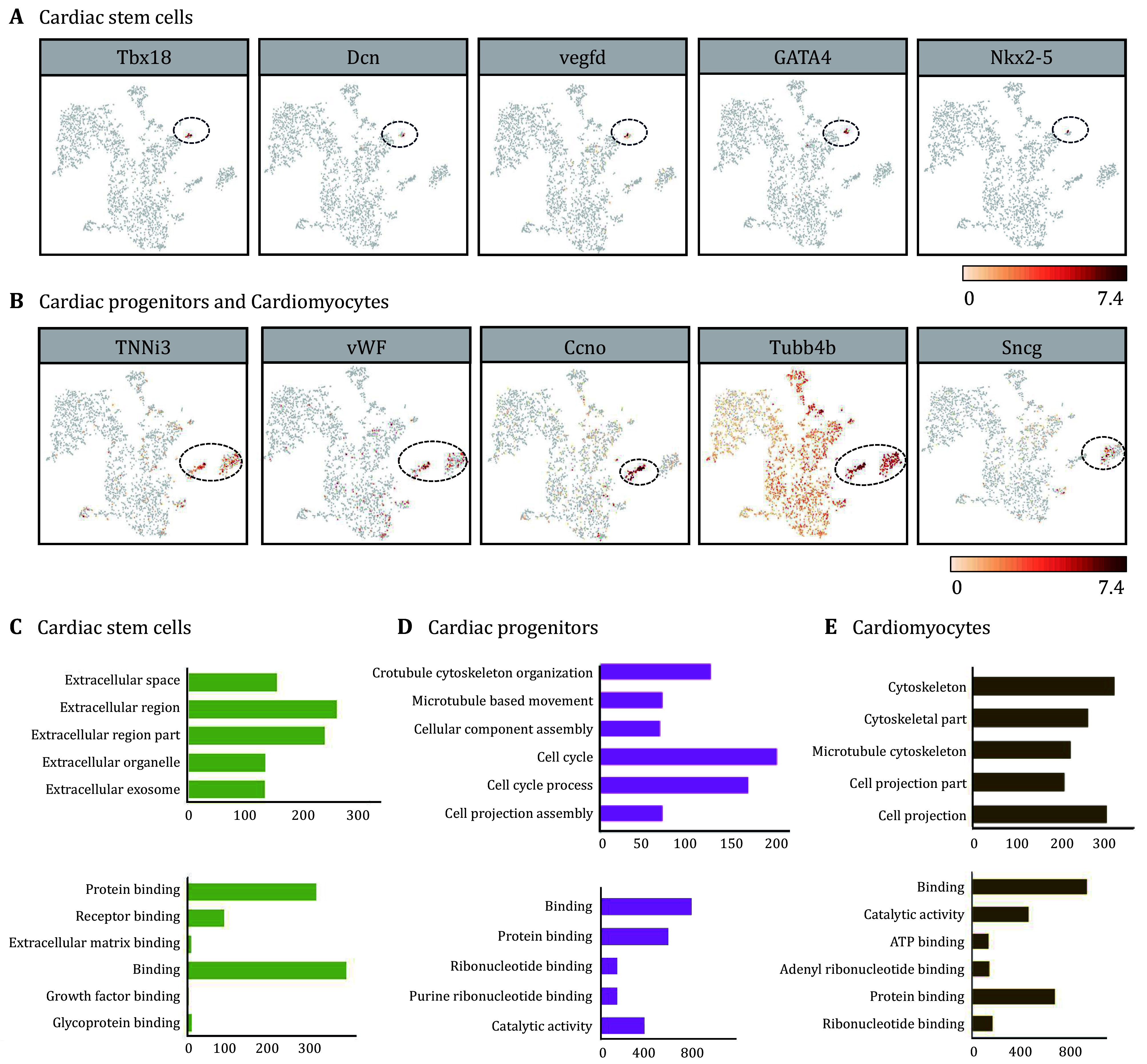
Recellularized construct contained cardiac-related cells. **A** The specific markers of cardiac stem cells were specifically expressed in c9. **B** The specific markers that were specifically expressed in cardiac progenitors (c6) and cardiomyocytes (c7). **C**-**E** GO analysis of the differently expressed genes in c9, c6 and c7. The section of the cellular component and molecular function (**C**) in c9; The section of biological process and molecular function (**D**) in c6; The section of cellular components and molecular function (**E**) in c7

C6, c7 and c9 were identified as cardiac lineage cells, which accounted for 8.20% of all cells. C9 was identified as cardiac stem cells with molecular signatures of Tbx18, Dcn, VEGFD, GATA4 and Nkx2-5 (Fig. 6A). GO analysis showed that differently expressed genes were enriched in the extracellular matrix and extracellular region in the cellular component category, and enriched in binding in the molecular function category (Fig. 6C). C6 and c7 were identified as cardiomyocytes and cardiac progenitors, respectively, and enriched with genes that encode cardiovascular markers, including TNNi3 and vWF (Fig. 6B). C7 was distinguished by the expression of canonical genes of proliferation and cell cycle, including Ccno, Kifc1 and MKi67 (Fig. 6B, supplementary Figs. S1 and S2). Remarkably, the cells displayed molecular signatures of immature neurons, including Tubb4b and sncg in c6 and c7, suggesting that the cardiac-like cells derived from NSCs partially maintained the signatures of neural lineage (Fig. 6B). The differently expressed genes in c6 and c7 were enriched in the cytoskeleton and cell cycle (Figs. 6D and 6E), implying the involvement in cytoskeleton organization and microtubule remodeling. In addition, the cells in c6 and c7 also showed an increased expression of genes for binding (Figs. 6D and 6E). These myocardial lineage cells have potentially important applications in myocardial regeneration. Due to the limited proliferative capacity of adult mammalian myocardial cells, myocardial cell transplantation and *in-situ* cell proliferation are focuses in the field of heart regeneration. C6 directly provides myocardial cells and C9 represents the proliferative state of myocardial cells capable of generating myocardial cells. Besides, C7 represents myocardial progenitor cells. Although they cannot directly regenerate myocardial cells, their paracrine function and ability to promote vascular regeneration are still crucial for cardiac repair. These studies suggested that the heart decellularized extracellular matrix may be an effective means of regulating cardiac regeneration.

### Cells originate from NSCs possess the dual signatures of both neural ectoderm and mesoderm during fate choices

**Figure 7:**
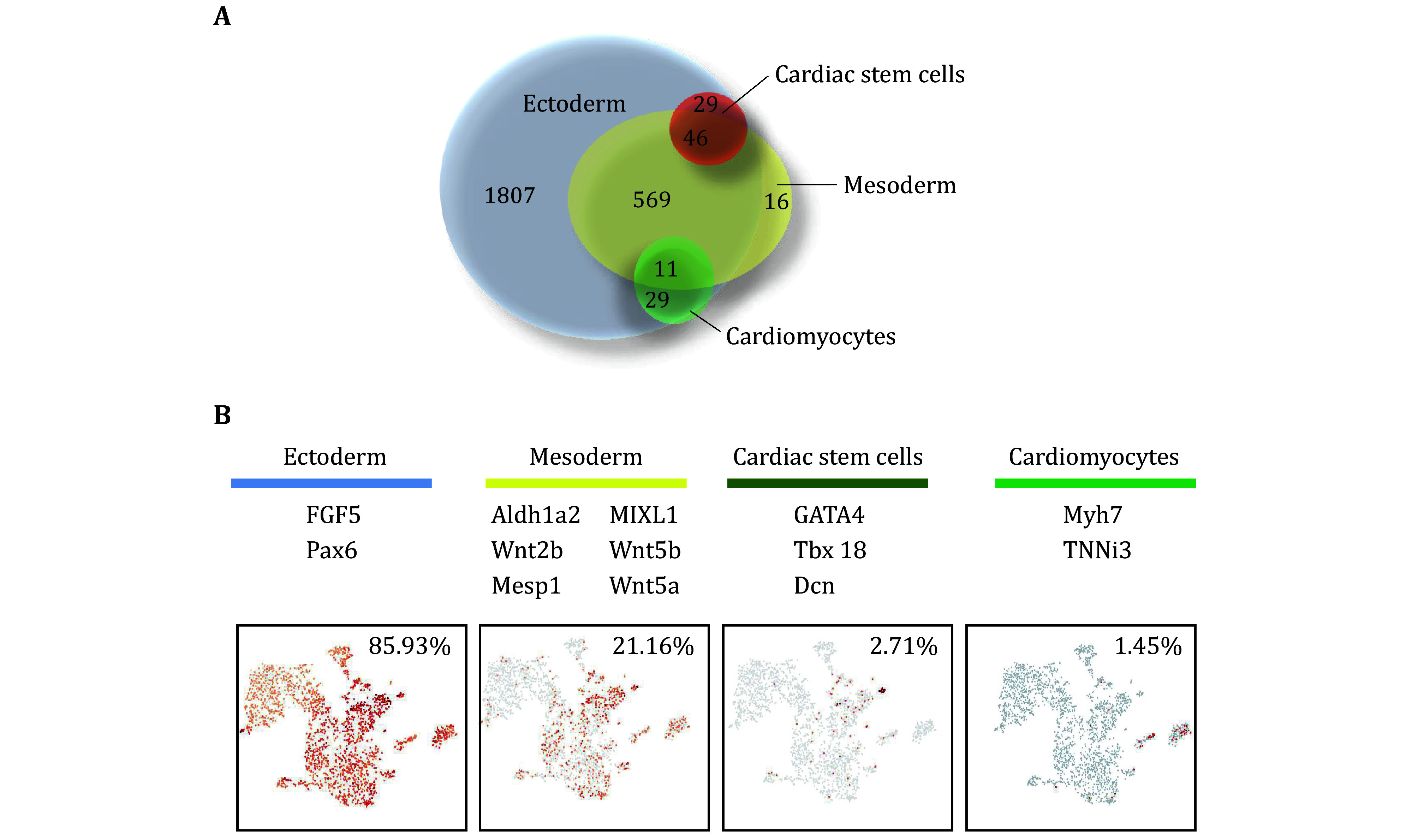
Refinement of promiscuous germ layer gene expression during fate choices. **A** Venn diagram representing the number of cells expressing germ layer or lineage specific genes. **B** tSNE plot of single-cell mRNA sequencing data containing neuroectoderm, mesoderm, cardiac stem cells and cardiomyocytes

We assigned the germ layer origin of cells by analyzing the expression of the specific markers (Fig. 7A). Hierarchical clustering of genes associated with mesoderm development, ectoderm development, cardiac stem cells and cardiomyocytes were specifically distributed on the *t*-SNE plot as shown in Fig. 7B. The results revealed that 2376 cells expressed the specific markers of ectoderm accounting for 85.93% of the total cells (2765 cells), of which 1807 cells were neural ectoderm origin, which accounted for 65.3% of all cells. 585 cells expressed the specific markers of mesoderm (21.16%), among which, 569 cells co-expressed the ectoderm specific markers (FGF5, Pax6), suggesting that these cells possessed the dual germ properties of both ectoderm and mesoderm. The number of cells expressing cardiac stem cells specific markers was 75 (2.71%), and 46 of them co-expressed the specific markers of mesoderm and ectoderm. 40 cells expressed the specific markers of cardiomyocytes (1.45%), among which 11 cells co-expressed the specific markers of mesoderm and ectoderm. These results suggested that the development processes of the cardiac-like cells derived from NSCs in the recellularized heart construct on day 14 were different from the normal cardiomyocyte in regard to the germ and lineage origin. They maintained the dual signatures of both neural ectoderm and mesoderm, which implied new cell states and changes in the cellular hierarchies across the germ layer take place with the development of recellularized constructs.

## DISCUSSION

In this study, to reveal the biological effects of heart decellularized matrix on cross-embryonic NSCs and the underlying mechanism, we seeded NSCs in heart decellularized matrix for seven days, and then analyzed 2765 cells by single-cell RNA sequencing. We found that the recellularized construct can achieve extensive development and generate an unprecedented diversity of cell types, including neurons, oligodendrocytes, astrocytes, neural stem cells, mature cardiomyocytes, immature cardiomyocytes, and cardiac stem cells.

NSCs have the potential to differentiate into neuron and glial cells, including astrocytes and oligodendrocytes, which form the basis of neurogenesis (McKay 1997). Previous studies have shown that the biochemical composition and physical properties of materials may influence cell commitment and differentiation of NSCs. Natural materials derived from extracellular matrices have good biocompatibility and biodegradability, supporting the growth, migration, proliferation, and differentiation of NSCs (Jain *et al*. 2020). Additionally, physical properties such as material stiffness and conductivity may impact cell fate. A stiffness of 1 kPa prefers to promote the maturation of neurons and the formation of neuronal networks compared to higher stiffness (Blaschke *et al*. 2019). Material conductivity may also modulate neuronal cell electrophysiology structurally and functionally, guiding the differentiation of neural stem cells. For example, carbon nanotube conductive materials promote neuronal differentiation and maintain neuronal network activity, which has been applied in preclinical studies of spinal cord regeneration, brain injury, and central nervous system damage (Nascimento *et al*. 2023; Ye *et al*. 2021). In this study, immunofluorescent staining revealed heart decellularized matrix reserved ECM components. Furthermore, single-cell sequencing analysis showed that cultured NSCs in heart decellularized matrix have the ability to differentiate into various neural cell types, including neurons, oligodendrocytes, and astrocytes. There existed a very small amount of undifferentiated NSCs, indicating complete differentiation of NSCs. Therefore, our data support heart decellularized extracellular matrix possessed endogenous ECM components and created a suitable microenvironment that supports NSC adhesion, growth, and differentiation.

Notably, single-cell sequencing analysis revealed that a small subset of NSCs differentiated into cardiac lineage cells, including cardiac stem cells, cardiomyocytes and cardiac progenitors. Importantly, the newly formed cell types of the cardiac lineage exhibited dual characteristics of both neural ectoderm and cardiac mesoderm (Fig. 7). These results confirmed that heart decellularized matrix promotes the transdifferentiation of cross-embryonic NSCs into the cardiac lineage, which is consistent with our earlier studies of TMT-based quantitative proteome profiles (Wang *et al*. 2021a). Many studies have demonstrated that iPSCs or fibroblasts can be reprogrammed into cardiomyocytes by transcription factors or small-molecule cocktail (Hashimoto *et al*. 2019; Wang *et al*. 2022). This suggests that the heart decellularized matrix possesses unique cardiac-specific factors or small molecules that influence the cell fate commitment of NSCs.

Previous studies have shown that NSCs can sense external mechanical changes in the microenvironment through integrins, mechanosensitive ion channels, G proteins, second messengers, and the cell cytoskeleton, and then convert mechanical signals into intracellular biological signals to alter the fate of NSCs (Chaudhuri *et al*. 2020; Martinac 2004). Consistently, in our earlier studies using tandem mass tag (TMT)-based quantitative proteome profiles to assess the impact of heart decellularized matrix on NSCs, we found enhanced ECM-receptor interactions and demonstrated that the interaction between cell surface integrins and integrin ligands in the ECM mediated the reprogramming of NSCs (Wang *et al*. 2021a). In this work, we compared the differentially expressed genes in each cluster. In the clusters of neural precursor cells (c5) and cardiac stem cells (c9) (Figs. 5D and 6C), differentially expressed genes were enriched in the extracellular region, implying their important role in cell-matrix interactions and regulation of responses to stimuli in the early stages of NSC differentiation. In the clusters of cardiac lineage cells (c6, c7, and c9), differentially expressed genes were enriched in the binding of the molecular function category in GO analysis (Figs. 6C–6E), which is consistent with previous studies indicating that binding processes can enhance the transmission of extracellular signals to the cytoplasm and strengthen cell-matrix interactions (Castillo *et al*. 2020; Luo *et al*. 2007). These results revealed parallel and divergent gene expression characteristics in the recellularized constructs. However, single-cell transcriptomic profiles at different differentiation times are needed to ultimately elucidate the specific differentiation processes of NSC in heart decellularized matrix.

## CONCLUSION

In conclusion, we investigated the trans-differentiation of NSCs in whole heart decellularized scaffold at single cell level and confirmed ECM highly contributes to NSCs heterogeneity. Importantly, cardiac-like cells derived from NSCs possessed the signatures of both neural ectoderm and cardiac mesoderm. These findings provide novel insight into tissue-specific cross-embryonic layer differentiation of stem cells, and pave the way for clinical applications of the decellularized matrix in cardiac tissue engineering.

## Conflict of interest

Xiaoning Yang, Yuwei Zhao, Wei Liu, Zhongbao Gao, Chunlan Wang, Changyong Wang, Siwei Li and Xiao Zhang declare that they have no conflict of interest.
